# The privacy protection of the internet of vehicles resource transaction details based on blockchain

**DOI:** 10.1371/journal.pone.0312854

**Published:** 2025-01-03

**Authors:** Jing Chen, Tao Li, Min Huang

**Affiliations:** 1 Yunnan Highway Network Toll Management Co., Ltd, Kunming, China; 2 Yunnan Key Laboratory of Digital Transportation, Kunming, China; 3 Yunnan Tengjian Technology Co., Ltd, Kunming, China; Jinan University, CHINA

## Abstract

The rapid development of Internet of Things technology has promoted the popularization of Internet of Vehicles, and its safety and reliability have become the focus of intelligent transportation system research. Vehicle-road collaboration relies on the collaborative computing and storage resources of the vehicle on-board unit (OBU), which are usually limited. When the vehicle in the edge area needs to do computing tasks such as intelligent driving, but its own computing resources are insufficient. Therefore, it needs other computing resources from idle vehicles and road side unit (RSU). This resource sharing can get additional computing resources to complete the task, and can be more convenient to complete the computing task quickly. Most current studies consider this type of resource sharing as a vehicle-to-vehicle (V2V) network transaction, aiming to stimulate the enthusiasm of vehicle sharing and optimize the utilization of computing resources in edge areas. However, the traditional blockchain transaction mode exposes serious privacy disclosure risks in vehicle networking resource transactions, including the openness and transparency of user identity, transaction details, and transaction addresses, which poses great challenges to data security. Therefore, this study innovatively proposed a blockchain-based privacy protection scheme for vehicle networking resource transaction details. By introducing committed value protection, zero-knowledge proof technology and constructing temporary transaction addressed mechanism. The scheme realized the comprehensive privacy protection of transaction funds, transaction details and transaction addresses, which could effectively avoid the disclosure of users’ sensitive information. Compared with the existing methods, the proposed scheme not only greatly enhanced the privacy protection capability, but also ensured the efficiency and security of transaction verification through zero-knowledge proof, avoiding the direct exposure of private keys. Meanwhile, the experimental verification demonstrates that the scheme not only enhances the level of privacy protection but also does not augment the supplementary processing burden. Furthermore, it is evident that the scheme meets the rigorous requirements for real-time resource transactions in the Internet of Vehicles.

## Introduction

With the rapid development and widespread adoption of Internet of Things (IoT) and smart mobile terminal technologies, the problem of personal information leakage has become increasingly serious, causing growing public concern [1,2]. Privacy protection in automotive network security services can be categorized into two main aspects: identity information privacy protection and data privacy protection, depending on the types of data to be protected [3–8]. The protection of identity information primarily includes personal data related to vehicles or users, such as user identification numbers and vehicle license plates. It also includes authentication information, such as relevant vehicle registration details. Data privacy in vehicle networking extends beyond typical transactional privacy to include business information generated by the various application scenarios. Despite extensive research focused on security services and strategies within the Internet of vehicles (IoV), the integration of blockchain technology with vehicle networking provides an effective means to address issues of data tampering and secure access during vehicle data transmission [9,10].

To improve the information exchange and safety issues between vehicles, many scholars have done related research. For example, Li et al. proposed a learning method in order to realize the vehicle-to-vehicle collaborative perception of loss communication. The results showed that this method could effectively improve the perception ability of vehicles in poor communication and promote vehicle information sharing in the edge area [11]. Noor-A-Rahim et al. analyzed enabling technologies, challenges, and opportunities to explore the application of 6G in vehicle to everything (V2X) communication. The studies revealed that 6G technology could greatly promote efficient resource sharing and communication between vehicles in the edge area [12]. Wang et al. designed an intelligent interconnected cruise control system that took into account vehicle-to-vehicle communication delays. The results showed that the system could still guarantee the cruise control performance under delayed conditions, which was conducive to the stable cooperation of vehicles in the edge area [13]. Son et al. proposed a lightweight vehicle-to-infrastructure (V2I) switching authentication protocol based on blockchain. The studies revealed that this protocol could improve the security of vehicle access network in edge areas and provide a trusted environment for resource sharing [14]. He et al. proposed a solution to the security and privacy challenges of vehicle digital twin networks. The results showed that these schemes could effectively ensure data security and privacy in the process of vehicle resource sharing in the edge area [15].

However, the high mobility of vehicles, the dynamic nature of network structures, and the presence of numerous heterogeneous end devices require low resource consumption and high processing efficiency in vehicle networking applications. In addition, the computing resources of most vehicles are limited. Traditional consensus mechanisms ensure blockchain fairness through computational power competition [16]. Existing research on transaction privacy protection mechanisms combined with blockchain technology typically focuses on achieving transaction non-repudiation using encryption and blockchain technology. However, it overlooks the transparency of blockchain transaction ledgers. Public transaction records can lead to association analysis attacks and reveal the private information of transaction participants [17]. Many studies associate user identity with public keys or other data representing identity, which poses a risk of privacy leakage. While attackers cannot directly obtain a user’s real identity information from the public key, they can infer privacy information from transaction ledgers and analyze transaction information patterns.

Transaction security based on blockchain technology should not only ensure transaction non-repudiation, but also address privacy protection mechanisms at each transaction stage. This study proposes a privacy protection scheme for vehicle network resource sharing services based on multi-chain collaborative hierarchical identification, which utilizes zero-knowledge proof technology to protect users’ funds, transaction details, and records in publicly accessible ledgers. In the resource sharing transaction process, to incentivize vehicle owners to share their idle computing resources, multiple users are allowed to participate in vehicle-to-vehicle (V2V) transmission. This study addresses the potential privacy leakage in shared resources with the following privacy protection requirements:

(1) Resource sharing transactions remain within the scope of blockchain transactions. Therefore, sensitive privacy information such as user identity, transaction details, and funds of participating buyers and sellers in V2V transactions must be desensitized to prevent attackers from mining transaction content and identifying user identities and account balances hidden in multiple related transactions through transaction accounting data analysis.

(2) Information such as the addresses and Merkle paths of the parties involved in resource-sharing transactions must also be protected to prevent attackers from performing correlation analysis and attacks using transaction addresses.

This study represents a significant contribution to the field of privacy protection in vehicle networking resource transactions, with a particular focus on addressing the risk of privacy disclosure in the existing blockchain transaction mode. By introducing commitment value protection and zero-knowledge proof technology, the research effectively protects the privacy of transaction funds and prevents the leakage of user account balance. At the same time, encryption and pseudorandom number generation technology are used to desensitize transaction details to prevent the disclosure of sensitive information. To prevent the transaction address association attack, a temporary transaction address mechanism is constructed and an n-to-many transmission mode is adopted to ensure the anonymity of both transaction parties. Furthermore, this study employs zero-knowledge proof technology to facilitate streamlined signature verification, thereby guaranteeing the legitimacy of the transaction while circumventing the direct exposure of the private key. Additionally, it considers the imperative for security and real-time functionality. Finally, the scheme not only improves the level of privacy protection, but also does not introduce additional processing delay, and realizes the efficient and secure operation of vehicle network resource transactions. In conclusion, the work presented in this study addresses the issue of privacy disclosure in blockchain transactions and simultaneously optimizes the transaction verification process through technological innovation. The result is a novel scheme that is secure, efficient, and offers robust privacy protection for vehicle networking resource transactions.

## Privacy protection scheme for transaction details

**Fig 1:**
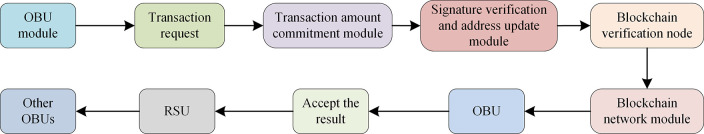
Overall scheme diagram proposed by the study.

In the realm of the IoV vehicles engaged in autonomous driving, vehicle-road coordination, and formation maneuvers require extensive computations based on roadside information, road conditions, and the positions of surrounding vehicles. In addition, when vehicles interact with each other, they must process and store the data they receive. However, the on-board computing units in today’s vehicles have limited computing and storage capabilities, often leading to resource constraints when vehicles perform multitasking computations. Currently, research predominantly adopts a strategy of offloading computation to the edge, using the idle resources of neighboring vehicles and roadside units to provide edge computing for vehicles with insufficient resources. Typically, this offloading strategy is defined as a transaction involving the sharing of computing resources among vehicles. While existing research focuses primarily on improving the efficiency of resource sharing, it overlooks two critical aspects: how to incentivize more networked vehicles to participate in sharing, and how to protect the privacy of such sharing. In addition, the widespread use of pseudonyms to ensure the privacy of sharing introduces complexities in management and storage, as well as security risks such as the disclosure of private information such as account balances and transaction associations. To address these challenges, this study proposes a computing resource sharing scheme based on a reverse auction mechanism to encourage greater participation of networked vehicles in resource sharing. The use of reverse auction algorithms not only incentivizes resource sharing participants, but also enables resource sharing sponsors to optimize their sharing costs. Moreover, to address the existing privacy protection challenges in resource sharing, a privacy protection mechanism for promised transactions is introduced by leveraging collaborative edge blockchain technologies. This mechanism ensures the privacy protection of transaction funds by transforming the data structure of blockchain transactions. In addition, the design includes account fund verification based on zero-knowledge proof to prevent the disclosure of user account information during the verification process. Through a transition between private keys and temporary transaction addresses, privacy protection of blockchain transaction addresses is achieved, culminating in a comprehensive privacy protection framework for blockchain-based resource sharing transactions. Finally, the study conducts security analyses and performance experiments on the proposed resource transaction auction algorithm and privacy protection scheme. The results demonstrate the effectiveness of the proposed algorithm and scheme in incentivizing vehicle resource sharing while providing robust transaction privacy protection. The overall scheme proposed by the research is shown in Fig 1.

In Fig 1, the overall scheme diagram shows the comprehensive process of privacy protection of vehicle networking resource transaction details based on blockchain. Through the registration and initialization module, the on-board unit (OBU) of the vehicle is registered and secured on the edge blockchain. In the transaction amount commitment module, both sides of the transaction cryptographically commit to the transaction amount to ensure the privacy of transaction funds. The signature verification and address update module uses zero-knowledge proof to verify transaction signatures and update temporary transaction addresses to ensure anonymity. The blockchain network module is responsible for the verification, storage, and consensus of all transactions to ensure transaction authenticity and immutability. Finally, the vehicle unit completes resource sharing and transactions through the blockchain network to achieve secure and efficient privacy protection of vehicle network resource transactions.

### Vehicle initialization

In the IoV system, due to the high mobility of vehicles and the dynamic structure of the network, the system adopts hierarchical management to improve the efficiency and flexibility of resource management. A lower tier, such as Tier 1, may cover a smaller geographic area, such as a city block or a section of a highway, and primarily handles direct communication and resource requests from vehicles in that area. As the layers ascend, the geographic area covered by the layers gradually expands, and the communication and resource requirements handled become more complex and diverse. The higher level (Layer 2, Layer 3, etc.) may be responsible for cross-regional resource scheduling, load balancing, and global communication optimization. During the vehicle initialization phase, in order for a vehicle within an edge area to engage in resource sharing service transactions within that area, it must first undergo registration within the edge area. By registering with the nearby edge blockchain, the vehicle receives secure identity verification, confirming its secure identity authentication within the edge domain. The specific registration process is outlined as follows:

1. When a vehicle’s OBU, which is located in the perception layer, transitions to an edge area network of the IoV, the vehicle must initiate registration with the edge blockchain within that edge area before conducting any transactions. During registration, the OBU undergoes private key extension protection provided by the security chain, completing the security identity authentication process. Only after successful authentication is the OBU allowed to join the edge chain.
  Assuming the OBU to be registered is situated within the *σ* layer of the edge area, with its identity denoted as *Uid*|_*σ*_, the upper chain verification nodes and the key security chain *Tskc*_*Uid*|*σ*−1_ of the edge chain are responsible for receiving and verifying its information. Upon successful verification, the OBU receives a private key protection enhancement factor $skc_{Uid{|}_{\sigma -1}}^{\left(i\right)}$. After successful registration verification, calculating ($mk_{Uid{|}_{\sigma }}^{\left(i,j\right)},B_{F}^{\prime },skc_{Uid{|}_{\sigma -1}}^{\left(i\right)}$) the complete private key $skc_{Uid{|}_{\sigma }}^{\left(i,j\right)}$ for the OBU located within *σ* layer is generated.
2. Upon completion of the OBU registration, the verification nodes of the edge blockchain reach a consensus to package the OBU’s information onto the blockchain. This signifies the successful registration of the OBU on the edge blockchain.

### Transaction amount commitment

**Fig 2:**
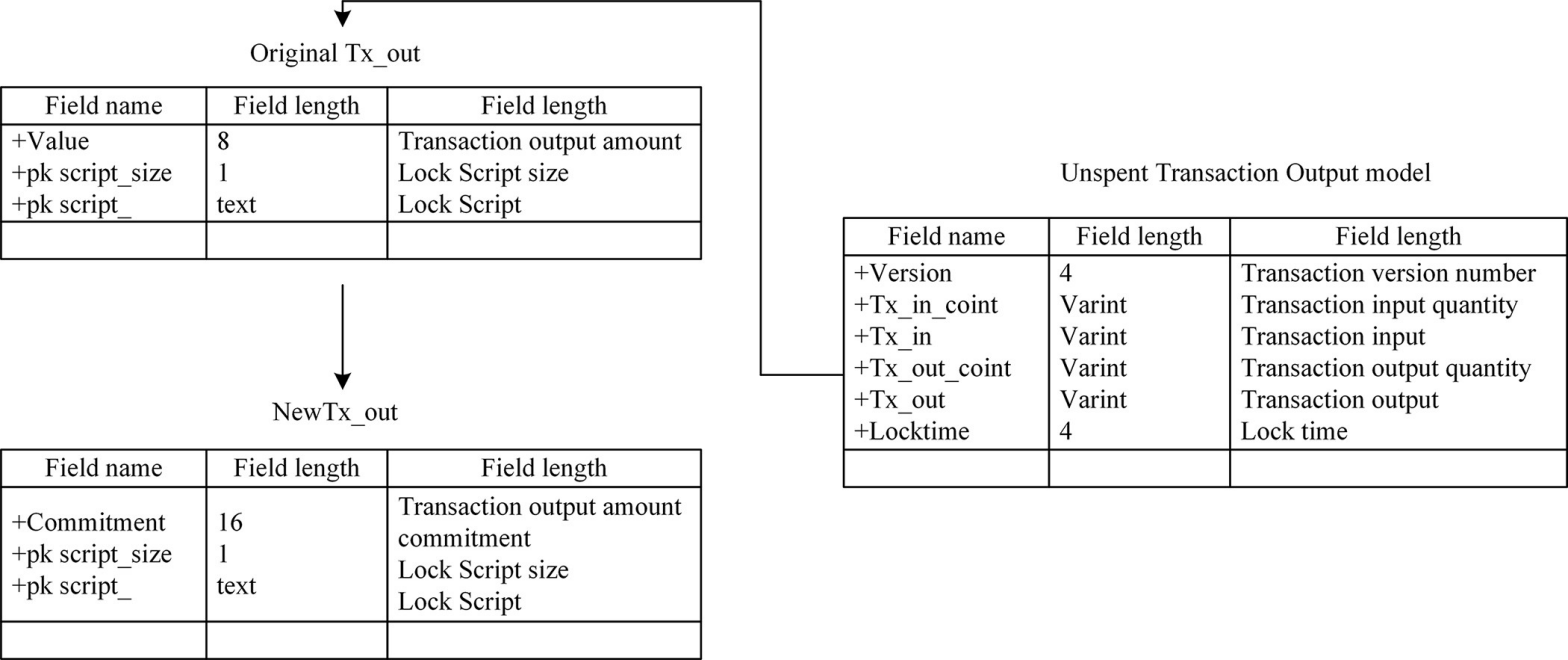
Blockchain transaction structure.

In the current blockchain transaction model, funds are stored in clear text and can be queried externally, making them vulnerable to acquisition by any potential adversary. To protect the financial privacy of both parties involved in V2V resource sharing transactions, this section describes the design of the blockchain transaction structure, as shown in Fig 2.

Fig 2 shows the design of the blockchain transaction structure, which is primarily used to protect the privacy of funds in V2V resource-sharing transactions. The privacy protection of transaction funds is achieved by replacing the transaction output funds and balances with commitments. Specifically, the transaction amount is no longer displayed directly in the transaction record, but is represented by encrypted commitment values, thus ensuring the privacy of the transaction amount. The design makes it impossible for attackers to directly obtain the transaction amount by querying the blockchain, and can only attempt to crack the promised value through complex mathematical calculations, greatly enhancing the security of privacy protection. At the same time, using zero-knowledge proof to verify transaction funds and balances can ensure the legality and non-repudiation of transactions without revealing any private information.

## Privacy protection of transaction amount

In this model, the blockchain is safeguarded by a root private key generator (PKG), m lower-level domain PKGs, n nodes’ random private keys, and public keys of *n*′ nodes.

Step 1: It involves generating protected blockchain verification chains (PRBVCs) for n verification nodes and private blockchain (PrvBC) for *n*′ verification nodes based on the input system security parameters and values *V*_*n*_≤m*n−*n*+1 determined by the root PKG. Additionally, it generates root PKG public parameters, root public and private keys, etc., for the 0th floor. The vehicle’s OBU queries the root PKG chain to obtain these parameters.

Step 2: It entails generating a private key composed of random private keys and hierarchical private keys. This process, facilitated by the collaboration of a key security chain and PKG from each domain, involves the key security chain generating a blinding factor, denoted as r, for the user. This factor is then sent to the user, who generates a commitment *Pcomm* = *rG*+*CoinH* for the transaction output amount.

Step 3: It involves generating a serial number using a pseudo-random number. This number is computed by combining the blinding factor and the private key. The first four bytes of this pseudo-random number serve as the unique identifier for the transaction.

**Algorithm 1. Transaction amount commitment algorithm.**

**Input**: *r* = *Random*(), *Coin* /*Amount

**Output**: ***PComm***/*Amount Commitment Value

1: Select_ECC(G,H);/*Generate an elliptic curve in a finite field and select points G and H.

2: ***Pcomm*** = ***rG***+***CoinH***;

3: Return ***Pcomm***

## Verification of transaction amount commitment

Let’s suppose vehicle A is within the edge area network, where multiple resource sharing service transactions occur among the vehicles in the area. If the quantity of the inputs for the *λ* transaction is k and the quantity of the outputs is t (*k*+*t*>1), with the real amount of the transaction inputs denoted as $Coin_{tx\_in}^{i}(i\in [1,k])$. The real amount of the transaction outputs is denoted as $Coin_{tx\_out}^{j}(j\in [1,t])$. Then the commitment values for the input and output of the first transaction are denoted as $Cmm_{tx\_in}^{i}$ and $Cmm_{tx\_out}^{j}$, respectively.

Thus, the expression is displayed as follows:

$$\begin{array}{l} Cmm_{tx\_in}^{i}=r_{tx\_in}^{i}G+Coin_{tx\_in}^{i}H\\ Cmm_{tx\_out}^{j}=r_{tx\_out}^{j}G+Coin_{tx\_out}^{j}H \end{array}$$ (1)

According to the additive homomorphism property and Pedersen’s commitment correctness, it’s necessary to verify $\sum_{i=1}^{k}Coin_{tx\_in}^{i}=\sum_{j=1}^{t}Coin_{tx\_out}^{j}$. The verification process is displayed as follows:

$$\begin{array}{l} \sum_{i=1}^{k}Coin_{tx\_in}^{i}=\sum_{j=1}^{t}Coin_{tx\_out}^{j}\Leftrightarrow \sum_{i=1}^{k}Coin_{tx\_in}^{i}-\\ \sum_{j=1}^{t}Coin_{tx\_out}^{j}=\sum_{i=1}^{k}\left(r_{tx\_in}^{i}G+Coin_{tx\_in}^{i}H\right)-\sum_{j=1}^{t}\left(r_{tx\_out}^{j}G+Coin_{tx\_out}^{j}H\right)\\ =(\sum_{i=1}^{k}r_{tx\_in}^{i}-\sum_{j=1}^{t}r_{tx\_out}^{j})G+(\sum_{i=1}^{k}Coin_{tx\_in}^{i}-\sum_{j=1}^{t}Coin_{tx\_out}^{j})H\\ =(\sum_{i=1}^{k}r_{tx\_in}^{i}-\sum_{j=1}^{t}r_{tx\_out}^{j})G \end{array}$$ (2)

Therefore, by introducing Pedersen commitment values for privacy protection of transaction output amounts, the transaction model can correctly verify transactions. The specific algorithm is displayed as follows::

**Algorithm 2. Transaction commitment value verification.**

**Input: $Coin_{tx\_in}^{i}(i\in [1,k])$, $Coin_{tx\_out}^{j}(j\in [1,t])$, $r_{tx\_in}^{i}$, $r_{tx\_out}^{j}$, *G***/*Public parameters

**Output: 1or 0** /*1 represents balance before and after, 0 represents imbalance.

1: while (i<k&&1< = i&&j<t&&j< = 1)do

2:  {   i++;

3:      j++;

4:    $d=\sum_{i=1}^{k}Coin_{tx\_in}^{i}-\sum_{j=1}^{t}Coin_{tx\_out}^{j}$;

5:    $e=\sum_{i=1}^{k}r_{tx\_in}^{i}-\sum_{j=1}^{t}r_{tx\_out}^{j}$;

6:  } end

7: if (d = = e*G)

8:  {Return 1;}

9:  else{ Return 0;}

### Signature verification

In the V2V mode of the resource sharing service, transaction processes need to be verified by scripts in the blockchain. To ensure the legitimacy of the transaction, it’s critical to validate the signatures of both parties. Signature verification requires the provision of the user’s public and private key pair. Therefore, to protect privacy during signature verification and prevent private key exposure, this section introduces a signature legitimacy verification method based on non-interactive zero-knowledge proof. This approach aims to mitigate private key exposure and prevent attackers from initiating fraudulent transactions masquerading as honest nodes to analyze and acquire private keys. The procedure is outlined below:

Step 1: Assume that the public and private key pair of the vehicle OBU is (*Obu*_*sk*_,*Obu*_*pk*_), and *Obu*_*pk*_ = *Obu*_sk_*G;

Step 2: The vehicle OBU first calculates $r=\sum_{i=1}^{n}r_{tx\_in}^{i}$, thus obtaining *R* = r*G;

Step 3: If the shared transaction record is *Info*_*s*_, then *e* = *hash*(*R*||*Info*_*s*_), *s* = *r*+e*sk;

Step 4: The verification node verifies the vehicle OBU’s message (*e*,*s*) according to the sum *Obu*_*pk*_ and G, i.e.,:

$$\begin{array}{l} R^{\prime }=s*G-e*Obu_{pk}\\ e^{\prime }=hash(R^{\prime }\|Info_{s}) \end{array}$$ (3)

If the verification is passed, it means that the zero-knowledge proof verification is successful. The specific algorithm is described as follows:

**Algorithm 3. Signature verification.**

**Input: *r* = *Random*(), *Obu***_***sk***_, *G* /* public parameters

**Output:** 1 or 0 /*1 indicates that the signature is correct, and 0 indicates that it is incorrect.

1:  R = r*G

2:   **String(*Info***_***s***_**)**/*Transaction information consists of transaction time, ID, sn, etc.

3:  ***e* = *hash*(*R*||*Info***_***s***_**)**

4:  *s* = *r*+e*sk

5:  $\begin{array}{l} R^{\prime }=s*G-e*Obu_{pk}\\ e^{\prime }=hash(R^{\prime }\|Info_{s}) \end{array}$6:  if (***e* == *e*′**)

7:  {Return 1;}

8:  else{ Return 0;}

### Transaction address update

In blockchain transactions, attackers can often perform correlation analysis by analyzing all transaction records associated with a public ledger, inferring relevant information between users and recipient addresses. To mitigate this correlation analysis, it is necessary to enhance the privacy protection method of transaction addresses, in accordance with the recommendations set forth in the preceding sections.

**Fig 3:**
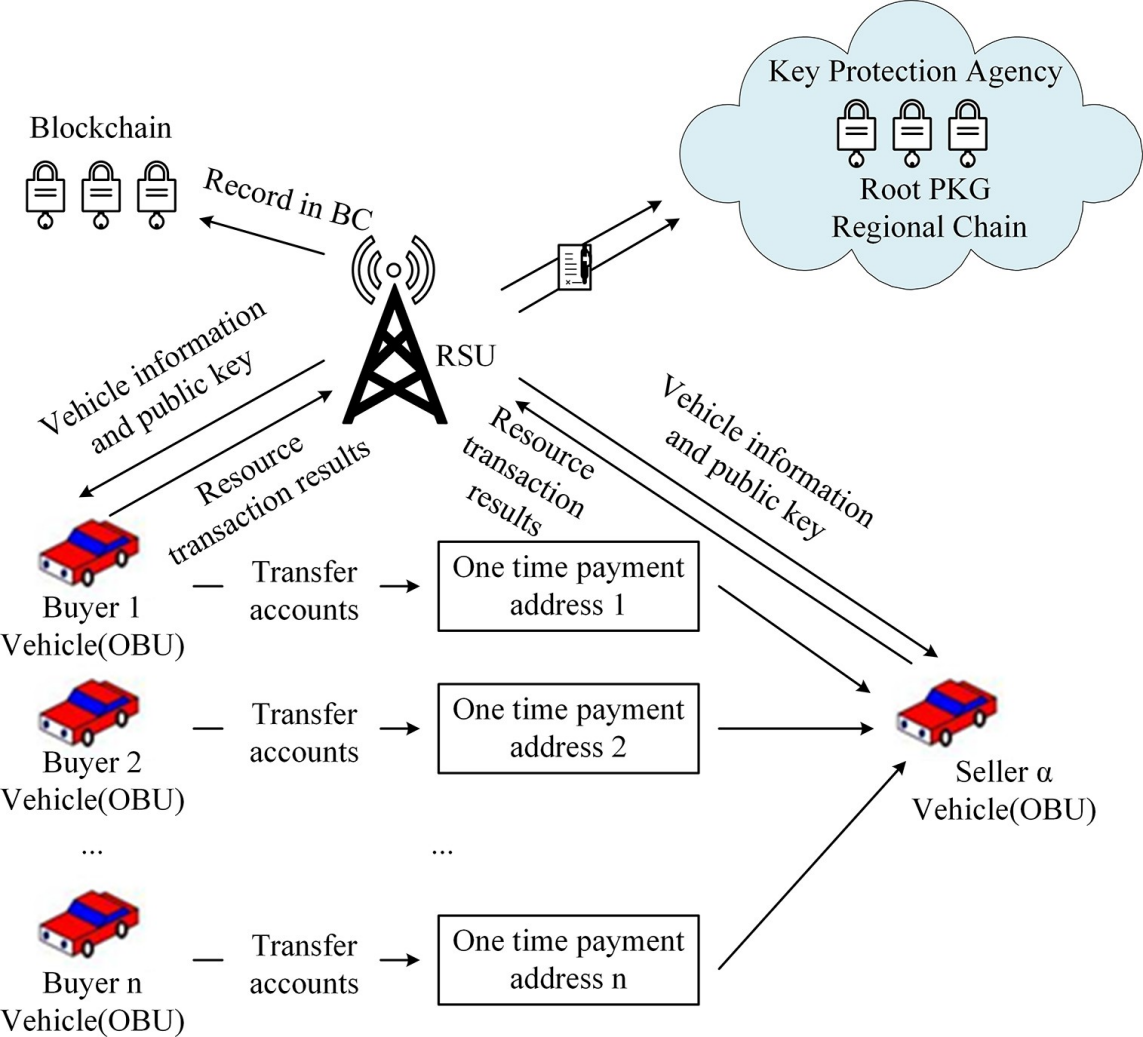
Transaction address generation.

In traditional blockchain transactions, users typically utilize a single transaction address. To prevent attackers from correlating and analyzing transactions associated with the same address, an improvement to the existing transaction receiving method is proposed. This improvement is based on the use of concepts from existing anonymity and obfuscation techniques. In this section, which addresses the scenario of V2V resource sharing services, it is proposed that an n-to-many transfer mechanism for payment addresses be adopted in order to enhance the privacy protection of recipient transaction addresses. The basic process is illustrated in Fig 3.

Fig 3 shows the transaction address generation process, which is mainly used to enhance the privacy protection of transaction addresses. The seller’s vehicle generates a temporary public key and a unique transaction identification serial number, which are signed and stored on the blockchain. This information is then encrypted and sent to the buyer’s vehicle, which decrypts it to generate the payment address and complete the transfer. The n-to-many temporary address transmission mechanism can effectively prevent attackers from obtaining user privacy through association analysis. Each transaction uses a different temporary public key and address, ensuring the anonymity and untraceability of the transaction. In addition, the authenticity and immutability of the transaction are guaranteed by the verification and storage of the blockchain. In Fig 2, the specific steps for generating transaction addresses are as follows:

Step 1: The seller’s vehicle *α*, acting as the payee, generates a temporary public key and a unique transaction identification serial number $d\alpha_{pk_{i}},i\in (1,\cdots n)$ for receiving payment. These are signed by *sk*_*α*_ and road side unit (RSU) submitted to the local blockchain verification node for validation. After validation, they are stored on the blockchain. Subsequently, they are encrypted with the public key of the transaction initiator and forwarded to the buyer’s vehicle to create an encrypted triplet $E_{{oubi}_{pk}}(sign_{sk_{\alpha }}(d\alpha_{pk_{i}}),d\alpha_{pk_{i}},sn_{obu_{i}})$.

Step 2: The buyer’s vehicle *Obu*_*i*_, acting as the transaction initiator, decrypts the received encrypted triplet using its own private key *sk*_*obui*_. After verification, it extracts the temporary public key $d\alpha_{pk_{i}},i\in (1,\cdots n)$ and generates the payment address for this transaction. It then proceeds to conduct the transfer operation to this address.

Step 3: The seller’s vehicle performs the transfer of funds to the temporary payment address *i*∈(1,⋯*n*).

## Security analysis

### Transaction address security

1. Identity concealment: The transaction address hiding scheme proposed in this chapter ensures the anonymity of the recipient of resource sharing transactions through n-to-many temporary address transfers. By packaging and signing the temporary public key through the blockchain at the edge, it prevents the generation of duplicate temporary addresses and facilitates third-party auditing. Since the temporary public key is used only once in each transaction, attackers cannot query and analyze the relevance of the transaction through open ledger records. The recipient can access funds using only his or her private key. Using the proxy transfer function of temporary addresses effectively hides the transfer relationship between the two parties involved in resource sharing transactions, ensuring their anonymity and preventing attackers from inferring users’ real information through transaction address association analysis.
2. Fund security: When the recipient needs to access funds from the temporary address, they only need to prove ownership using zero-knowledge proof. After successful verification, they can use their private key to access the funds in the address. Attackers who do not have access to the actual private key cannot pass the zero-knowledge verification and therefore cannot prove ownership of the funds. As a result, they are unable to access the funds, ensuring the security of the funds.

### Security of transaction fund commitments

The security of transaction fund commitments relies on the difficulty of the discrete logarithm problem and is characterized by the following aspects:

1. Prevention of commitment value forgery
  Assuming that the commitment value of transaction funds is *Pcomm* = *rG*+*CoinH*. If the blinding factor *r* is leaked, the probability of a malicious attacker being able to calculate the funds within a limited time can be effectively ignored. If a malicious attacker attempts to forge a commitment value of the same amount, it is denoted as *r*′,*Coin*′, then ∃*r*′ and ∃*Coin*′. Its expression is showed as follows:
$$\begin{array}{l} Pcomm=rG+CoinH=r^{\prime }G+Coin^{\prime }H\\ \Rightarrow (r-r^{\prime })G=(Coin^{\prime }-Coin)H\Rightarrow G={\left(r-r^{\prime }\right)}^{-1}(Coin^{\prime }-Coin) \end{array}$$ (4)
  Since *Pcomm* = *rG*+*CoinH*, the discrete logarithm problem is known to be difficult. Moreover, malicious attackers cannot find a solution to satisfy this equation within polynomial time. Hence, successfully forging the commitment value is infeasible.
2. Completeness
  As verifiers, blockchain verification nodes within the edge area can legitimately assess the input and output commitment values of transactions, as well as the blinding factors, through a consensus mechanism, making them tamper-proof.
3. External audit
  If external auditing is required to evaluate the legality and completeness of transactions within the system, one can first compute the value based on the publicly available G, H values, random number R, and Coin. Subsequently, this computed value *Pcomm* = *rG*+*CoinH* can be compared with the existing commitment value. In case of deceitful transactions between the transaction initiator and the node, the values would not match.

### Signature verification security

The signature verification proposed in this section is based on zero-knowledge proof. Below is an analysis of the security of signature verification.

1. Non-forgery of the private key
  Assuming the prover forges *Obu*_*sk*_′, a random number is *r*′, then the formula is expressed:
$$\begin{array}{l} R^{\prime }=r^{\prime }*G\Rightarrow \left\{ \begin{array}{c} e^{\prime }=hash(R^{\prime }\|Info_{s})\\ s^{\prime }=r^{\prime }+e^{\prime }*Obu_{sk} \end{array}\right.\\ \;\Rightarrow ZK(e^{\prime },s^{\prime }) \end{array}$$ (5)
  If the verifier receives *ZK*(*e*′,*s*′), then the formula is expressed:
$$\begin{array}{c} ZK(e^{\prime },s^{\prime }),R^{\prime \prime }=s^{\prime }*G-e^{\prime }*Obu_{pk}=(r^{\prime }+e^{\prime }*Ob{u^{\prime }}_{sk})G-e^{\prime }*Obu_{pk}\\ =R^{\prime }+e^{\prime }*Ob{u^{\prime }}_{sk}*G-e^{\prime }*Obu_{pk}\neq R^{\prime } \end{array}$$ (6)
  It is evident from *R*″⁠≠*R*′ that the prover cannot successfully forge *e* by utilizing the collision resistance of the hash function after forging *Obu*_*sk*_′, hence the prover cannot successfully counterfeit the private key proof.
2. Protection of private key information
  During the zero-knowledge proof process, the prover reveals only the public key and public parameters in the verification process. When sending evidence to the verifier, it is imperative not to reveal the private key and random number R. In essence, no secrets belonging to the prover are revealed to the verifier, thus ensuring privacy protection throughout the verification process. In the proposed privacy scheme, zero-knowledge proof is used to verify the signature of the transaction vehicle. Throughout this verification process, neither the vehicle nor the RSU can access the private key *Obu*_*sk*_ of the transaction initiator. Consequently, attackers posing as verifiers cannot recover the private key *Obu*_*sk*_ of the prover through zero-knowledge evidence *ZK*(*e*,*s*).

## Experiment and result analysis

The experiments are conducted on a server with an Intel Xeon Platinum 8269C 2.5GHz CPU*4 and 32GB of memory, running the CentOS7 operating system. Python, Zokrates open-source tools. Moreover, the Ethereum DApp development simulator are utilized.

### Scheme security analysis

The security of the proposed privacy protection for resource sharing is analyzed by comparing it with several existing blockchain schemes. The security of identity authentication methods, transaction funds, and transaction addresses are compared and analyzed. Table 1 depicts the details.

**Table 1:** Comparative analysis of scheme safety.

Scheme	Identity security	Transaction amount	Transfer relationship
Scheme 1[18]	CA certificate	Not hidden	Hidden
Scheme 2[19]	CA certificate	Not hidden	Hidden
This study	Identity identification	Hidden	Hidden

Compared with Scheme 1 and Scheme 2, the scheme proposed by the study adopts identity identification for identity security, which effectively avoids the problems associated with traditional certificate management. In terms of transaction amount, the scheme proposed by the study uses a promise hiding mechanism, which provides users with a superior method to protect the privacy of transaction funds, while Scheme 1 and Scheme 2 do not support transaction amount hiding. With respect to the transaction payment address, the scheme proposed by the study uses temporary addresses and transit modes to hide the relationship between the transaction address and the identity of the transaction vehicle, providing enhanced privacy protection for identity information. Scheme 1 and Scheme 2 can only conceal the payment address. Furthermore, the scheme introduced in this chapter utilizes zero-knowledge proof to verify the authenticity of transaction signatures, thereby enhancing both the security of resource sharing transactions and the privacy protection of trading vehicles.

### Time consumption of zero-knowledge signature verification

In Scheme 1, transaction information needs to be signed using a private key certificate first and then verified using a public key. This chapter proposes a zero-knowledge proof method for signature verification. To evaluate the time cost of zero-knowledge proof signature verification in detail, a comparative experiment is conducted. The experiments are performed on a CentOS7 server equipped with an Intel Xeon Platinum 8269C 2.5GHz CPU (quad-core) and 32GB of memory, utilizing the Python programming language combined with the Zokrates open source tool and the Ethereum DApp development simulator. In the experiment, several sets of transaction information of different lengths and corresponding public and private key pairs are generated as test data to ensure the completeness and reliability of the results. Then, the traditional signature verification scheme and the zero-knowledge proof-based signature verification scheme are compared and tested. In each test group, the total signature generation and verification times of the two schemes are recorded, and the experiment is repeated 8 times for each data group to calculate the average time and reduce random errors. The experimental results show that the signature verification scheme based on zero-knowledge proof is significantly better than the traditional scheme. The traditional scheme (Scheme 1) requires frequent public key encryption and private key decryption in the signature generation and verification process, resulting in a long time, and the signature verification time is 58.6 ms. In contrast, although the generation process of zero-knowledge proofs is more complex and requires some computational resources, the validity of the signature can be quickly confirmed in the verification phase by the proof alone. This is due to the fact that its verification process does not require access to the private key information, thus greatly reducing the verification time. The signature verification time is 22.5ms. In light of a comprehensive examination of the entire process of signature generation and verification, it can be concluded that the signature verification scheme based on a zero-knowledge proof exhibits superior time efficiency and practical application potential.

### Processing performance

**Fig 4:**
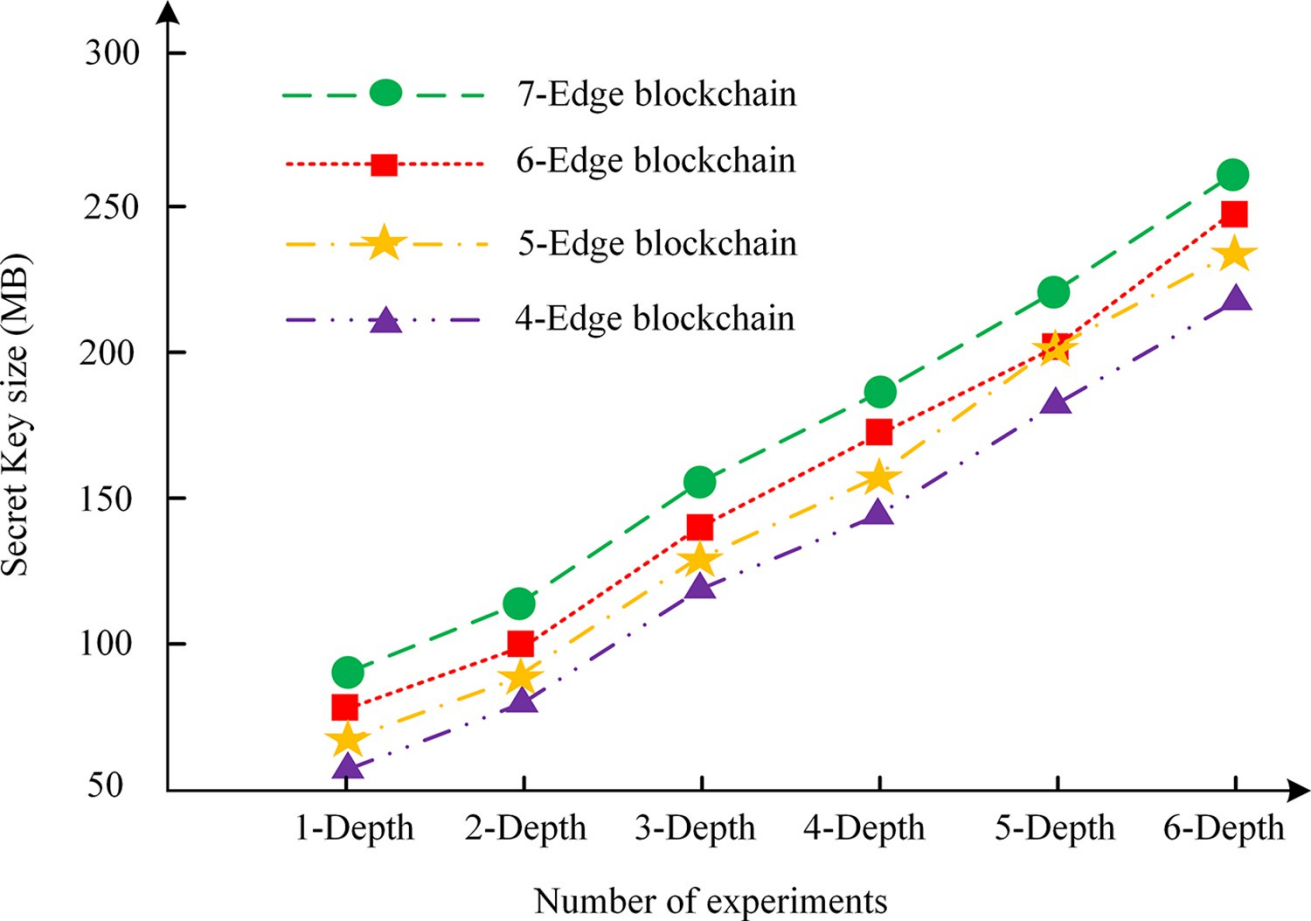
Growth of key.

**Fig 5:**
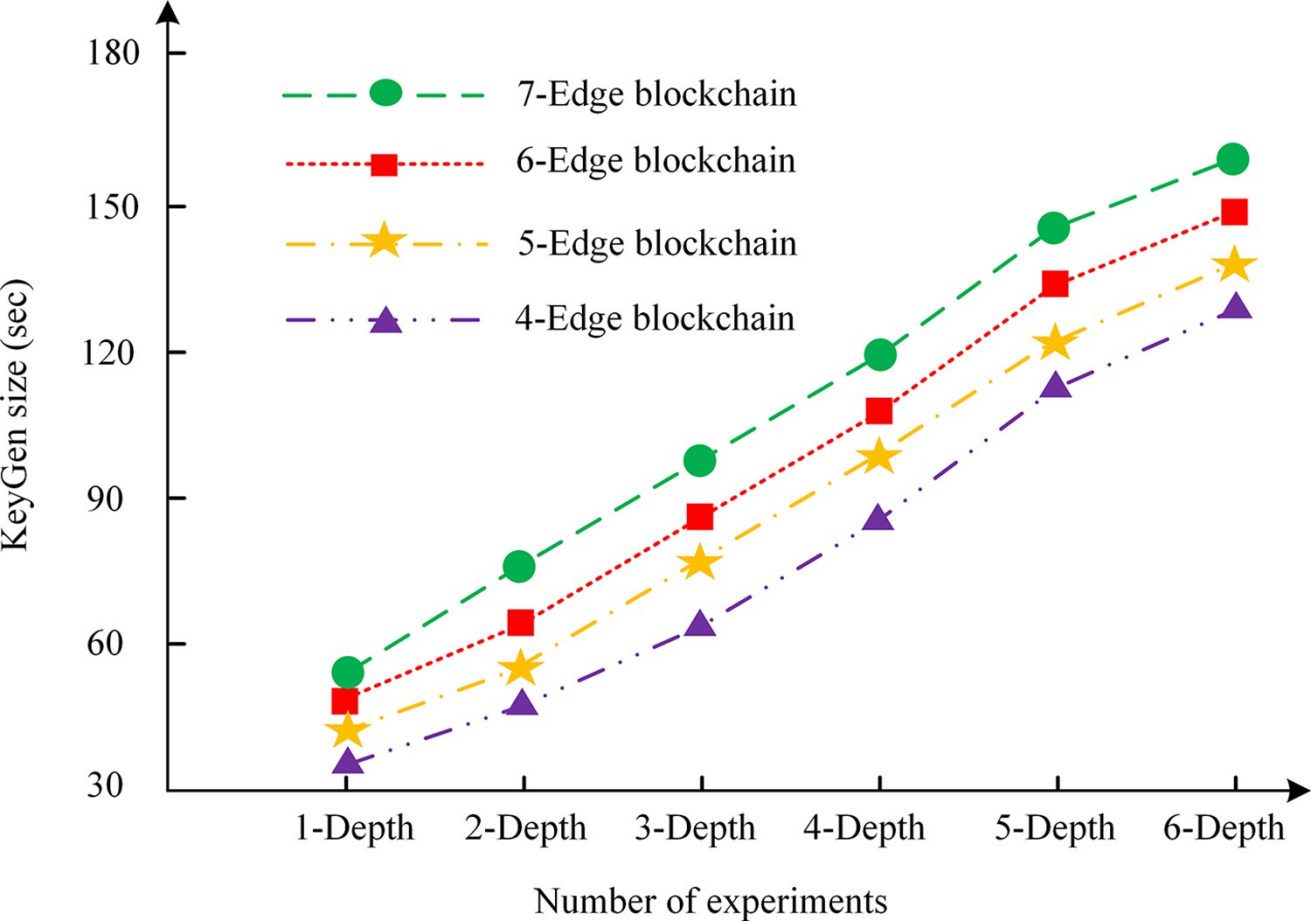
Key generation time.

**Fig 6:**
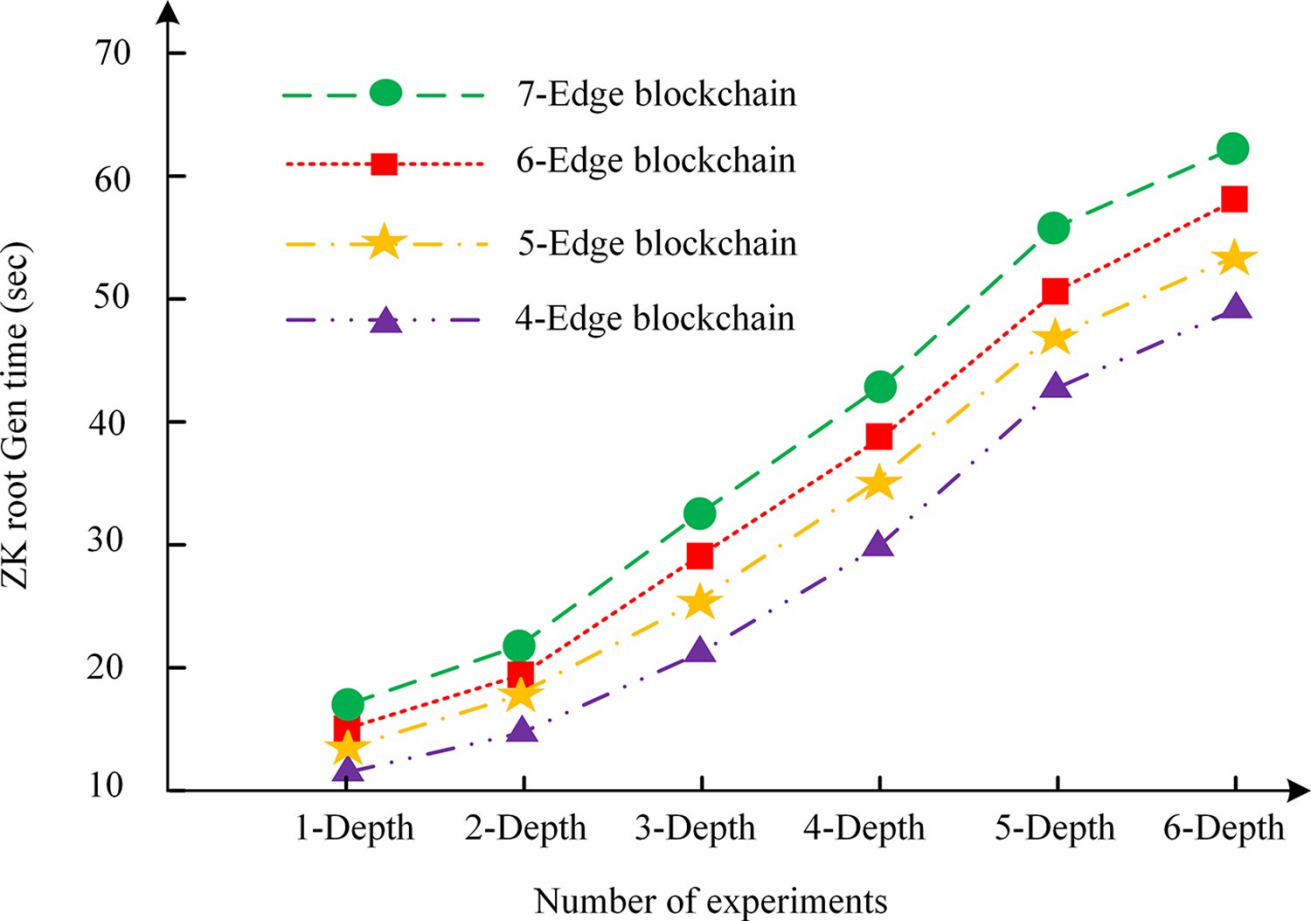
Zero-knowledge proof generation time.

To ascertain the efficacy of the privacy protection proposed for V2V in this chapter and to evaluate the system performance under conditions of high transaction volume, an experimental analysis is conducted on the key generation capacity, the time required for the generation of vehicle key pairs, and the time required for the generation of zero-knowledge proofs. Its objective is to exam the impact of different layers and different numbers of verification nodes in the edge area chain of the vehicle network. The results are shown in Figs 4–6.

In Figs 4–6, 4–edge blockchain, 5-edge blockchain, 6-edge blockchain, and 7-edge blockchain indicate that the number of blockchain edge networks is 4, 5, 6, and 7, respectively. In addition, in Figs 4–6, "depth" refers to the number of layers in the IoT or the number of edge area networks. As the scale of the vehicle network increases, the number of edge area networks also increases, which affects the size of the key, the key generation time, and the zero-knowledge proof generation time. The greater the depth, the greater the number of blockchain nodes and data to be processed, and therefore the greater the demand for computing resources.

According to the analysis in Fig 4, as the depth of the IoV network increases, the number of keys and the amount of data on the blockchain will also increase significantly. As a result, the key size will increase, which will require more storage space and processing power. The reason for this outcome may be that the introduction of each new element to the edge area network gives rise to the necessity for additional key generation and storage requirements, thereby increasing the complexity of the blockchain.

In Fig 5, the key generation time increases linearly with the increase of depth. The underlying cause of this outcome may be attributed to the fact that key generation is a highly computationally demanding process, necessitating the use of sophisticated encryption algorithms and random number generation. As the network grows in size, the number of these tasks also increases.

In Fig 6, the generation time of zero-knowledge proofs also increases linearly with the increase of depth. The reason for this result may be attributed to the inherent complexity of a zero-knowledge proof, which necessitates the resolution and validation of numerous mathematical problems. As the volume of transactions increases, so does the computational burden of zero-knowledge proof. It can be concluded that the key size, key generation time and zero-knowledge proof generation time of the system are affected by the number of different edge area networks. As the number of edge area networks increases, so does the size of the corresponding edge blockchain, resulting in a linear increase in key size, key generation time, and zero-knowledge proof generation time.

## Conclusion

In this study, the privacy protection of blockchain-based IoV resource sharing service is discussed in depth, and a comprehensive privacy protection scheme is proposed. The study employed a committed value protection mechanism to safeguard the confidentiality of transaction funds, thereby preventing potential attackers from acquiring information about the transaction amount through the public ledger. This approach was utilized to analyze the pivotal aspect of privacy disclosure in the context of vehicle networking resource sharing transactions. At the same time, by introducing zero-knowledge proof technology, the security of the private key and the privacy of the transaction verification were ensured while verifying the transaction signature. In addition, the temporary address generation and transmission mechanism was proposed, which effectively prevented attackers from inferring the real identities of transaction participants through association analysis. The experimental results showed that the proposed privacy protection scheme did not introduce significant processing delay when processing large transactions, which verified the feasibility and high efficiency of the scheme. Moreover, through comparison and analysis with existing schemes, the proposed scheme showed significant advantages in identity authentication, transaction amount protection, and transaction address privacy protection. In summary, the proposed privacy protection scheme of blockchain-based vehicle network resource sharing service has made significant progress in protecting the privacy and security of transaction participants. This achievement not only helps promote the wide application of vehicle network resource sharing services, but also provides important theoretical support and practical reference for the application of blockchain technology in intelligent transportation systems. In the future, it is necessary to continue to optimize and improve the scheme to cope with the more complex and changing network environment and privacy protection needs.

## Supporting information

S1 Appendix Symbol and corresponding meaning. (DOCX)

## References

[1] Yao Y, Shu F, Li Z, Cheng X, Wu L. Secure transmission scheme based on joint radar and communication in mobile vehicular networks. IEEE transactions on intelligent transportation systems, 2023, 24(9): 10027–10037.

[2] Hussein N H, Yaw C T, Koh S P, Tiong S K, Chong K H. A comprehensive survey on vehicular networking: Communications, applications, challenges, and upcoming research directions. IEEE Access, 2022, 10(3): 86127–86180.

[3] Tripathi K N, Yadav A M, Sharma S C. TREE: trust-based authenticated and secure dissemination of emergency event information for the network of connected vehicles. Arabian Journal for Science and Engineering, 2022, 47(8): 10689–10717.

[4] Wang J, Liu J. Secure and reliable slicing in 5G and beyond vehicular networks. IEEE Wireless Communications, 2022, 29(1): 126–133.

[5] Yao Y, Zhao J, Li Z, Cheng X, Wu L. Jamming and eavesdropping defense scheme based on deep reinforcement learning in autonomous vehicle networks. IEEE Transactions on Information Forensics and Security, 2023, 18(5): 1211–1224.

[6] Zhao T, Yurtsever E, Paulson J A, Rizzoni G. Formal certification methods for automated vehicle safety assessment. IEEE Transactions on Intelligent Vehicles, 2022, 8(1): 232–249.

[7] Ju Z, Zhang H, Li X, Chen X, Han J, Yang M. A survey on attack detection and resilience for connected and automated vehicles: From vehicle dynamics and control perspective. IEEE Transactions on Intelligent Vehicles, 2022, 7(4): 815–837.

[8] Yang S, Tan J, Lei T, Linares-Barranco B. Smart traffic navigation system for fault-tolerant edge computing of internet of vehicle in intelligent transportation gateway. IEEE transactions on intelligent transportation systems, 2023, 24(11): 13011–13022.

[9] Qureshi K N, Sandila M A S, Javed I T, Margaria T, Aslam L. Authentication scheme for unmanned aerial vehicles based internet of vehicles networks. Egyptian Informatics Journal, 2022, 23(1): 83–93.

[10] Waheed A, Shah M A, Mohsin S M, Khan A, Maple C, Aslam S, et al. A comprehensive review of computing paradigms, enabling computation offloading and task execution in vehicular networks. IEEE Access, 2022, 10(1): 3580–3600. doi: 10.1109/ACCESS.2021.3138219

[11] Li J, Xu R, Liu X, Ma J, Chi Z, Ma J, et al. Learning for vehicle-to-vehicle cooperative perception under lossy communication. IEEE Transactions on Intelligent Vehicles, 2023, 8(4): 2650–2660. doi: 10.1109/TIV.2023.3260040

[12] Noor-A-Rahim M, Liu Z, Lee H, Khyam M O, He J, Pesch D, et al. 6G for vehicle-to-everything (V2X) communications: Enabling technologies, challenges, and opportunities. Proceedings of the IEEE, 2022, 110(6): 712–734.

[13] Wang Z, Jin S, Liu L, Fang C, Li M, Guo S. Design of intelligent connected cruise control with vehicle-to-vehicle communication delays. IEEE Transactions on Vehicular Technology, 2022, 71(8): 9011–9025.

[14] Son S, Lee J, Park Y, Das A K. Design of blockchain-based lightweight V2I handover authentication protocol for VANET. IEEE Transactions on Network Science and Engineering, 2022, 9(3): 1346–1358.

[15] He C, Luan T H, Lu R, Su Z, Dong M. Security and privacy in vehicular digital twin networks: Challenges and solutions. IEEE Wireless Communications, 2022, 30(4): 154–160.

[16] Wang B, Su R. A distributed platoon control framework for connected automated vehicles in an urban traffic network. IEEE Transactions on Control of Network Systems, 2022, 9(4): 1717–1730.

[17] Ahed K, Benamar M, Lahcen A A, El Ouazzani R. Forwarding strategies in vehicular named data networks: A survey. Journal of King Saud University-Computer and Information Sciences, 2022, 34(5): 1819–1835.

[18] Liu Z, Wan L, Guo J, Huang F., Feng X., Wang L., et al. PPRU: A privacy-preserving reputation updating scheme for cloud-assisted vehicular networks. IEEE Transactions on Vehicular Technology, 2023.

[19] Guo J, Li X, Liu Z, Ma J., Yang C., Zhang J, et al. TROVE: A context-awareness trust model for VANETs using reinforcement learning. IEEE Internet of Things Journal, 2020, 7(7): 6647–6662.

