# Supplementary material for: The privacy protection of the internet of vehicles resource transaction details based on blockchain
Source: PLoS One. 2025 Jan 3;20(1):e0312854. doi: 10.1371/journal.pone.0312854 (PMC11698359; doi:10.1371/journal.pone.0312854)
Supplement: S1 Appendix — (DOCX) [file pone.0312854.s001.docx]

**Appendix**

**Appendix 1.** Symbol and corresponding meaning

| Symbol | Description |
| --- | --- |
| *G* | The selected base point on the elliptic curve used to generate public and private key pairs |
| *H* | Another point on the elliptic curve, together with the base point G, is used to generate the key |
| *r* | Blinding factor, used to generate commitment value, protect transaction amount privacy |
| *OBU* | On-board Unit - the communication and control unit On the vehicle |
| *A* | Vehicle A involved in the transaction |
| *B* | Another vehicle or RSU (Roadside Unit) involved in the transaction |
| *k* | The number of transactions entered |
| *t* | The number of output transactions |
| *x* | The actual amount of the transaction entered |
| *y* | The actual amount of the transaction output |
| *Cx​* | The committed value of the transaction input amount |
| *Cy​* | The committed value of the transaction output amount |
| *R* | A random number used to generate a transaction serial number |
| *d* | One of the intermediate values calculated during transaction verification |
| *e* | The intermediate value of the transaction verification process |
| *G′* | In signature verification, the calculated point |
| *sn* | The serial number of the transaction, which uniquely identifies the transaction |
